# Integration of Circulating Tumor DNA and Metabolic Parameters on ^18^F‐Fludeoxyglucose Positron Emission Tomography for Outcome Prediction in Unresectable Locally Advanced Non‐Small Cell Lung Cancer

**DOI:** 10.1002/advs.202413125

**Published:** 2025-03-16

**Authors:** Leilei Wu, Zhenshan Zhang, Chenxue Jiang, Li Li, Xiaojiang Sun, Menglin Bai, Ming Liu, Kangli Xiong, Jinbiao Shang, Jinming Yu, Shuanghu Yuan, Yang Yang, Yaping Xu

**Affiliations:** ^1^ Department of Radiation Oncology Shanghai Pulmonary Hospital School of Medicine Tongji University Shanghai 200433 China; ^2^ Department of Thoracic Surgery Shanghai Pulmonary Hospital School of Medicine Tongji University Shanghai 200433 China; ^3^ Department of Radiation Oncology Zhejiang Cancer Hospital Hangzhou 310022 China; ^4^ Department of Radiation Oncology The First Affiliated Hospital of USTC Division of Life Sciences and Medicine University of Science and Technology of China Hefei Anhui 230001 China; ^5^ Department of Radiation Oncology Anhui Provincial Cancer Hospital Hefei Anhui 230031 China; ^6^ Department of Radiation Oncology Shandong Cancer Hospital and Institute Shandong First Medical University and Shandong Academy of Medical Sciences Jinan Shandong 250117 China; ^7^ Department of Radiation Oncology Qilu Hospital of Shandong University Jinan Shandong 250012 China; ^8^ Geneseeq Research Institute Nanjing Geneseeq Technology Inc Nanjing 210008 China; ^9^ Department of Thyroid Surgery Zhejiang Cancer Hospital Hangzhou 310022 China; ^10^ Central Laboratory, Shanghai Pulmonary Hospital, School of Medicine Tongji University Shanghai 200433 China; ^11^ School of Materials Science and Engineering Tongji University Shanghai 201804 China

**Keywords:** ctDNA, definitive chemoradiotherapy, immune checkpoint inhibitors, LA‐NSCLC, PET metabolic parameters

## Abstract

This prospective study explores the prognostic value of circulating tumor DNA (ctDNA) and positron emission tomography/computed tomograpy (PET/CT) in unresectable locally advanced non‐small cell lung cancer (LA‐NSCLC) treated with definitive chemoradiotherapy (CRT). The discovery set includes 62 patients, with 62 baseline and 53 post‐CRT plasma samples. PET/CT is performed at baseline, and 33 patients undergo mid‐treatment scans after 40 Gy. Baseline ctDNA is detected in 71.0% of patients. Pre‐treatment ctDNA concentration correlates with total metabolic tumor volume (TMTV) (*p *< 0.001) and total lesion glycolysis (TLG) (*p *= 0.001) but not treatment response or survival. However, patients with undetectable ctDNA and low TMTV show significantly longer progression‐free survival (PFS) (34.2 vs 10.1 months, *p *= 0.027). Post‐CRT, ctDNA is detected in 47.2% of patients, while ctDNA concentration (*p *= 0.005) and variant allele frequency (VAF) (*p *= 0.005) significantly decline. Undetectable post‐CRT ctDNA associates with longer PFS (*p *< 0.001) and overall survival (OS) (*p *= 0.001). Higher ∆TMTV correlates with improved PFS and OS. Similar findings were obtained in a test of 19 patients. These results highlight post‐CRT ctDNA and ∆TMTV as robust prognostic markers, potentially identifying patients who may forgo ICI consolidation.

## Graphical abstract

This study highlights the combined predictive ability of ctDNA and PET parameters. Specifically, in the current “less is more” era of integrated therapy, combining post‐treatment ctDNA and ∆TMTV can guide consolidation therapy decisions, potentially avoiding unnecessary ICI treatment for certain patients. Future multi‐omics integration will further enhance precision treatment guidance.

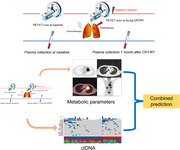

## 1 Introduction

Lung cancer remains the foremost cause of cancer death worldwide, with non‐small cell lung cancer (NSCLC) constituting 80–90% of cases.^[^ ^1^ ^]^ About one‐third of NSCLC patients have unresectable locally advanced (LA) disease at diagnosis, traditionally treated with definitive chemoradiotherapy (CRT), yielding a modest overall survival (OS) rate of ≈20%.^[^ ^2^, ^3^ ^]^ The advent of immune checkpoint inhibitors (ICI) consolidation following CRT has markedly reshaped the treatment landscape for unresectable LA‐NSCLC.^[^ ^4^, ^5^ ^]^ However, there's a pressing need for reliable biomarkers to predict CRT efficacy and guide individualized ICI consolidation post‐CRT.

Circulating tumor DNA (ctDNA), derived from tumors and present in peripheral blood as part of plasma cell‐free DNA (cfDNA), can be tracked through liquid biopsies to monitor treatment responses.^[^ ^6^ ^]^ Detection of molecular residual disease (MRD) via ctDNA post‐curative treatment indicates progressive disease in various cancers.^[^ ^7^, ^8^, ^9^ ^]^ Increasing studies are examining the clinical value of ctDNA detection in unresectable LA‐NSCLC, suggesting that post‐CRT ctDNA levels could predict CRT efficacy and guide subsequent ICI consolidation treatment.^[^ ^7^, ^10^, ^11^, ^12^ ^]^ However, the prognostic role of baseline ctDNA remains unclear.

On the other hand, ^18^F‐fluorodeoxyglucose (FDG) positron emission tomography/computed tomography (PET/CT) emerges as a preferred modality for staging, treatment response assessment, and radiation therapy (RT) regimen planning in NSCLC.^[^ ^13^ ^]^ Moreover, the employment of ^18^F‐FDG PET/CT has indeed ameliorated the prognosis of NSCLC patients.^[^ ^14^ ^]^ Several studies have investigated its predictive role in treatment responses and survival outcomes of definitive CRT for LA‐NSCLC, yet a consensus has not been attained.^[^ ^13^, ^15^, ^16^, ^17^ ^]^ In conjunction with ctDNA, it may potentially augment predictive accuracy, particularly at baseline.

Herein, we conducted a prospective, non‐interventional, observational study to delineate the role of ctDNA detection prior to and following definitive CRT/RT in patients with unresectable stage III NSCLC. Specifically, the prognostic utility of baseline and mid‐treatment ^18^F‐FDG PET/CT was also explored, aiming to establish a more robust predictive model.

## 2 Results

### 2.1 Patient Characteristics and Baseline ctDNA Analysis

**Figure 1:**
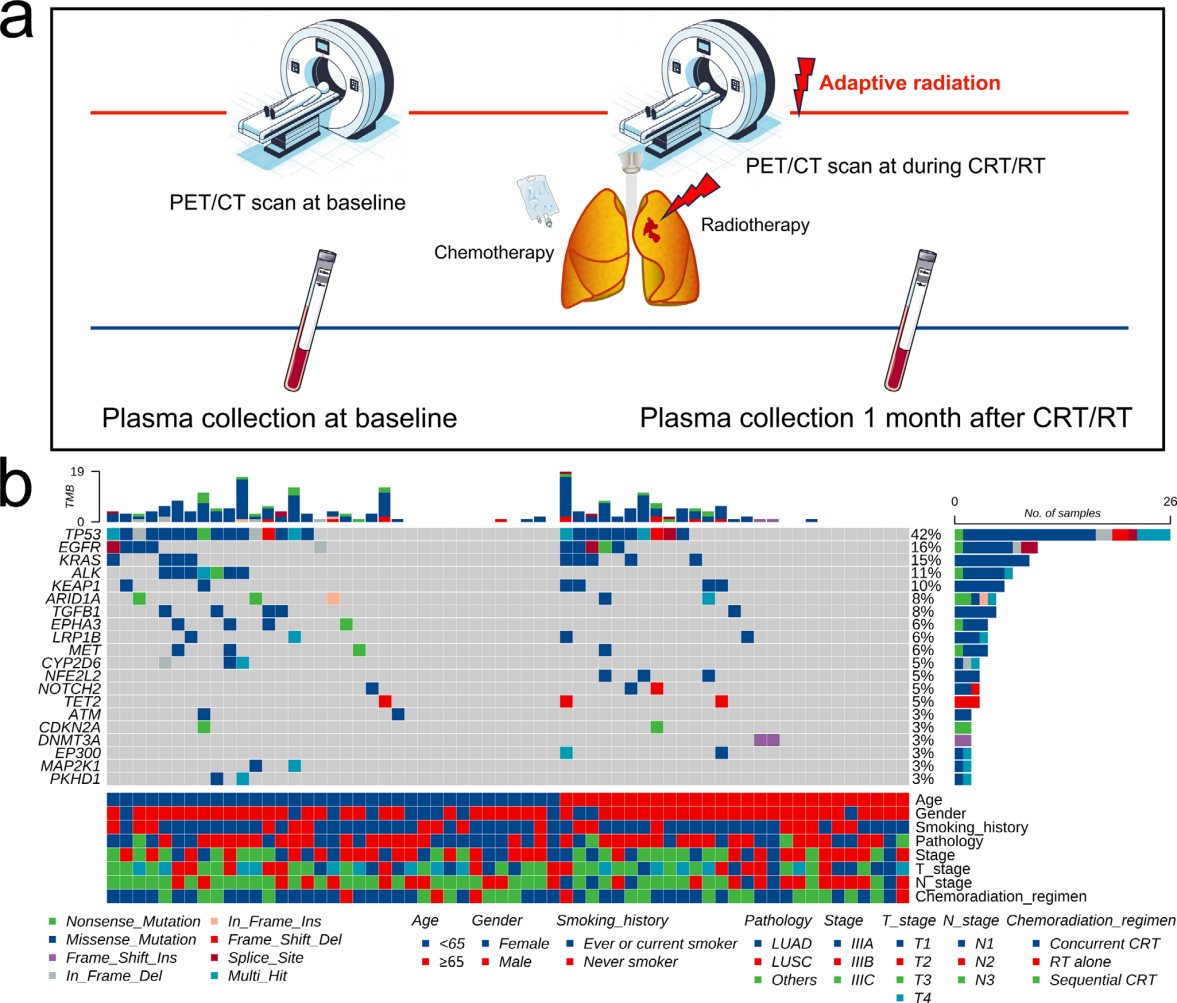
Study Diagram and Baseline. a) Study diagram of the study. b) Heatmap visualization reflecting each patient's baseline characteristics and pretreatment ctDNA assessments. Abbreviations: CRT, chemoradiotherapy; RT, radiotherapy; LUAD, lung adenocarcinoma; LUSC, lung squamous cell carcinoma.

**Figure 2:**
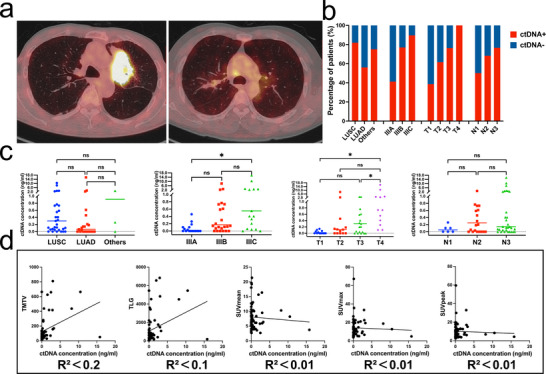
Comparative analysis of ctDNA among different baseline characteristics. a) Representative images of high, low metabolism in ^1^ ^8^F‐FDG PET/CT. b) Comparative analysis of ctDNA detection across different baseline characteristics. c) Comparison of baseline ctDNA concentration among different baseline characteristics. d) Linear regression analysis of baseline ctDNA concentration, metabolic parameters. Abbreviations: LUAD, lung adenocarcinoma; LUSC, lung squamous cell carcinoma; TMTV, total metabolic tumor volume; TLG, total lesion glycolysis; SUVmean, mean standardized uptake value; SUVmax, maximum standardized uptake value; SUVpeak, peak standardized uptake value.

In this study, a total of 62 patients were included in the discovery set, with plasma samples collected at baseline for ctDNA analysis. One month after completing CRT/RT, a second ctDNA detection was conducted in 53 patients. The process of definitive CRT, PET scanning, and plasma collection is shown (**Figure** **1**a). The average age of the participants was 63.4 years (ranging from 42 to 83 years), with 9 females and 44 patients (71.0%) having a history of smoking. The diagnosis of NSCLC was histologically validated in all patients via biopsy, including 33 (53.2%) cases of lung squamous cell carcinoma (LUSC), 25 (40.3%) of lung adenocarcinoma (LUAD), and 4 (6.5%) of unspecified histological subtypes. Baseline staging was carried out based on imaging information, categorized 17 (27.4%) patients as stage IIIA, 26 (41.9%) as IIIB, and 19 (30.6%) as IIIC, respectively (**Table** **1**). The baseline characteristics of 19 patients in the test set are also summarized (Table 1), which show comparable demographic and clinical features to the discovery set. The comprehensive pretreatment assessment of all patients using ^18^F‐FDG PET/CT revealed an average maximum standardized uptake value (SUVmax) of 14.01 (standard deviation [SD] = 9.76) and a maximum mean standardized uptake value (SUVmean) of 21.39. We present representative images of high and low metabolism in ^18^F‐FDG PET/CT from two different patients (**Figure** **2**a). Detailed imaging information of these two patients (Figure S1b,c, Supporting Information), as well as PET images of another patient before and after treatment, are also presented (Figure S1a, Supporting Information).

**Table 1:** Patient baseline characteristics.

Characteristics	Discovery set		Test set	
	Total (*n* = 62)	Detectable ctDNA (*n* = 44)	Total (*n* = 19)	Detectable ctDNA (*n* = 12)
Age				
Median	64 (42–83)	64 (42–83)	60 (48–78)	61.5 (53–78)
Mean ± SD	63.4 ± 8.8	62.9 ± 9.7	61.9 ± 8.3	63.7 ± 8.5
<65	35 (56.5)	27 (61.4)	12 (63.2)	7 (58.3)
≥65	27 (43.5)	17 (38.6)	7 (36.8)	5 (41.7)
Gender				
Male	53 (85.5)	37 (84.1)	16 (84.2)	10 (83.3)
Female	9 (14.5)	7 (15.9)	3 (15.8)	2 (16.7)
Smoking history				
Never smoker	18 (29.0)	13 (29.5)	12 (63.2)	4 (33.3)
Ever or current	44 (71.0)	31 (70.5)	7 (36.8)	8 (66.7)
Histology				
LUSC	33 (53.2)	27 (61.4)	8 (42.1)	5 (41.6)
LUAD	25 (40.3)	14 (31.8)	10 (52.6)	6 (50.0)
Missing data	4 (6.5)	3 (6.8)	1 (5.3)	1 (8.3)
Stage				
IIIA	17 (27.4)	7 (15.9)	6 (31.6)	4 (33.3)
IIIB	26 (41.9)	20 (45.5)	7 (36.8)	5 (41.7)
IIIC	19 (30.6)	17 (38.6)	6 (31.6)	3 (25.0)
T stage				
T1	13 (21.0)	5 (11.4)	2 (10.5)	1 (8.3)
T2	13 (21.0)	8 (18.2)	5 (26.3)	3 (25.0)
T3	21 (33.9)	16 (36.4)	6 (31.6)	4 (33.3)
T4	15 (24.2)	15 (34.1)	6 (31.6)	4 (33.3)
N stage				
N1	6 (9.7)	3 (6.8)	1 (5.3)	1 (8.3)
N2	22 (35.5)	15 (34.1)	8 (42.1)	6 (50.0)
N3	34 (54.8)	26 (59.1)	10 (52.6)	5 (41.7)

Abbreviations: SD, Standard deviation; LUSC, lung squamous cell carcinoma; LUAD, lung adenocarcinoma.

At baseline, ctDNA was detected in 44 patients (71.0%), with an average of 5.9 mutations per patient. The median cfDNA concentration across all patients stood at 12.97 ng mL^−1^, with a maximum value of 180.15 ng mL^−1^ (Figure 1b). The median variant allele frequency (VAF) of patients detected was 2.20%, with ctDNA concentrations ranging from 0.03 to 15.82 ng mL^−1^. In the test set of 19 patients, 12 patients (63.2%) had positive baseline ctDNA (Table 1), with the maximum ctDNA concentration being 4.31 ng mL^−1^. A comparative analysis among different baseline characteristics showed that patients with detectable baseline ctDNA tended to have more advanced disease, and the differences were significant across various stages (*p *= 0.004) and T stages (*p *= 0.003) (Figure 2b). Further analysis of ctDNA concentrations was carried out across various stages, T stages, N stages, and pathological types (Figure 2c). Significant differences were also observed among different stages (IIIA vs IIIC, *p *= 0.025) and T stages (T3 vs T4, *p *= 0.047; T1 vs T4, *p *= 0.019) (Figure 2c), but not among N stages, suggesting that ctDNA detection may be more closely related to tumor size. A higher detection rate of ctDNA was noted in LUSC compared to LUAD (*p *= 0.032), with no significant difference in ctDNA concentration. We attempted a simple linear fit between baseline ctDNA concentration and metabolic parameters (Figure 2d). However, all R^2^ values were too small, indicating poor fit. We further investigated the correlation between baseline ctDNA concentration and metabolic parameters. None of these variables followed a normal distribution, and the results revealed that a strong correlation was observed between pretreatment ctDNA concentration and total metabolic tumor volume (TMTV) (Spearman rho = 0.480, *p *< 0.001) and total lesion glycolysis (TLG) (Spearman rho = 0.427, *p *= 0.001).

### 2.2 Integration of ctDNA Detection and TMTV Enhanced Baseline Predictive Value

**Figure 3:**
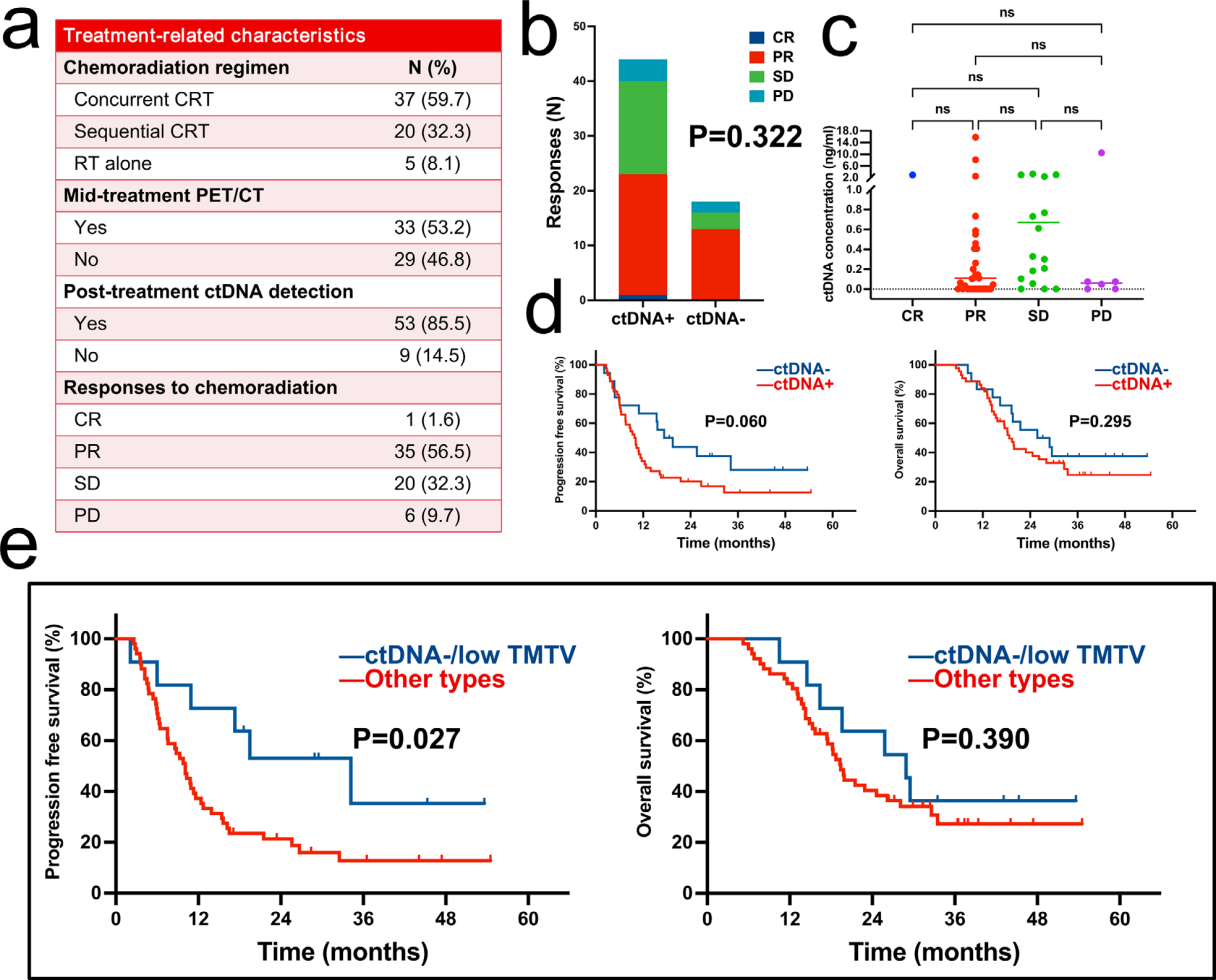
Treatment‐related characteristics and predictive value of integration of ctDNA status and TMTV for response and survival. a) Treatment‐related characteristics. b) Responses assessment based on baseline ctDNA status. c) Baseline ctDNA concentrations in patients with varying treatment responses. d) Kaplan–Meier curves for PFS and OS stratified by baseline ctDNA status. e) Kaplan–Meier curves for PFS and OS, comparing ctDNA‐/TMTV low group with other types. Abbreviations: CRT, chemoradiotherapy; ctDNA, circulating tumor DNA; CR, complete response; PR, partial response; stable disease; PD, progressive disease; TMTV, total metabolic tumor volume; TLG, total lesion glycolysis.

Given the crucial role of baseline information in guiding initial clinical decisions, we investigated the prognostic value of baseline ctDNA analysis. All 62 patients underwent curative dose RT, accompanied by concurrent (37, 59.7%) or sequential (20, 32.3%) chemotherapy, or without chemotherapy (5, 8.1%). Post‐treatment response evaluation revealed one patient achieved complete response (CR), 35 achieved partial response (PR), 20 achieved stable disease (SD), and 6 were assessed as progressive disease (PD), with treatment‐related information summarized (**Figure** **3**a). The baseline ctDNA detection and responses showed no significant association (*p *= 0.322, Figure 3b), and there was no significant difference in baseline ctDNA concentration among patients with different responses (Figure 3c). Similar findings were observed when comparing responders and non‐responders (*p *= 0.096). Moreover, baseline ctDNA status failed to differentiate patients with different prognoses, whether in terms of progression‐free survival (PFS) or OS, albeit with a certain trend in PFS differences (18.4 months vs 10.0 months, HR = 0.541, 95% CI: 0.282–1.037, *p *= 0.060; Figure 3d). Considering the correlation between pre‐treatment PET metabolic parameters and ctDNA detection, along with their potential predictive values, we combined ctDNA detection and PET metabolic parameters. We categorized parameters into high and low groups by median split dichotomization. All four metabolic parameters failed to effectively predict treatment responses. However, patients with ctDNA‐/low TMTV exhibited significantly better PFS (34.2 months vs 10.1 months, HR = 0.295, 95% CI: 0.126–0.694, *p *= 0.027; Figure 3e). Concurrently, pre‐treatment PET parameters themselves also did not show significant prognostic value in univariate analysis (low vs high, Table S1, Supporting Information). We further performed stratification based on baseline characteristics through subgroup analysis, and we found that pre‐treatment ctDNA‐/low TMTV was associated with longer PFS in patients aged ≥65 years (*p *= 0.029, HR = 0.244) and in those with LUAD (*p *= 0.036, HR = 0.204) (Figure S2, Supporting Information). The data from the test set also confirmed that the combined use of two modalities at baseline enhances prognostic ability. Three patients with ctDNA‐/low TMTV had significantly longer PFS, and all were alive at the last follow‐up. In contrast, neither pre‐treatment ctDNA nor PET parameters could effectively predict survival (Figure S3, Supporting Information). Therefore, integrating ctDNA detection and PET metabolic parameters enhanced the prognostic value of baseline characteristics.

### 2.3 Post‐Treatment ctDNA MRD and Changes before and after Treatment Were Highly Predictive of Recurrence and Survival

**Figure 4:**
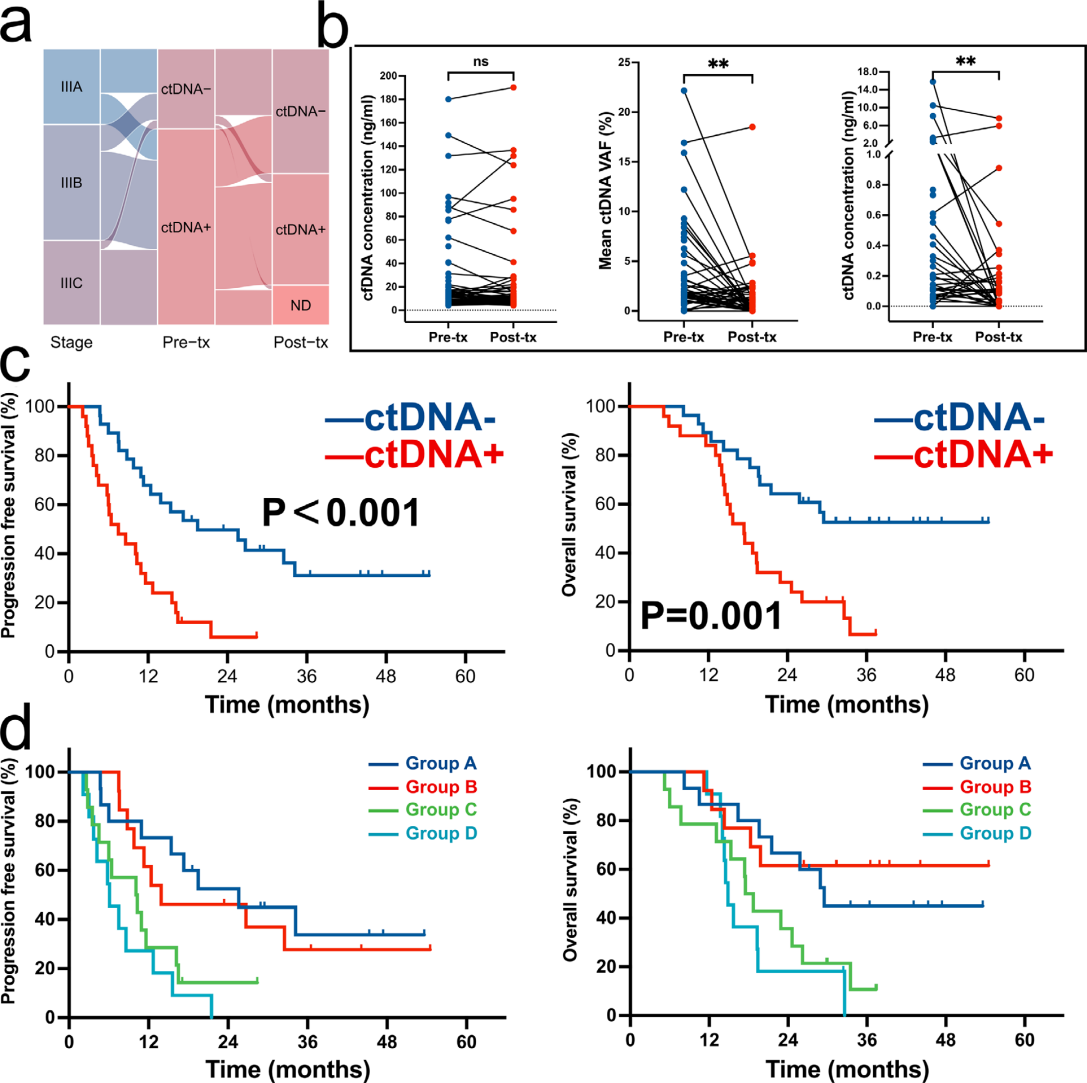
ctDNA changes following treatment and predictive value of ctDNA‐MRD post‐treatment. a) Sankey diagram illustrating the shifts in patient proportions, stratified by ctDNA status in pre‐treatment and post‐treatment time points. b) Changes of cfDNA, mean ctDNA VAF, and ctDNA concentration following treatment. c) Kaplan–Meier curves for PFS and OS stratified by post‐treatment ctDNA status. d) Kaplan–Meier curves for PFS and OS further stratified by pre‐ and post‐treatment ctDNA status. Abbreviations: ctDNA, circulating tumor DNA; VAF, variant allele fraction; tx, treatment; PFS, progression‐free survival; OS, overall survival; ns, not significant; *p < 0.05; **p < 0.01.

Following this, we further investigated the prognostic significance of ctDNA at one month following treatment. Excluding the nine patients who did not undergo ctDNA testing post‐treatment, ctDNA was detected in 25 of the remaining patients following treatment. We proceeded to classify the patients further based on the alterations in ctDNA status before and after treatment: 15 were consistently negative for ctDNA pre‐ and post‐treatment, designated as Group A; 13 were positive pre‐treatment but cleared post‐treatment, designated as Group B; 14 were positive pre‐treatment with reduced but not cleared ctDNA levels post‐treatment, designated as Group C; and 11 had not only no decrease but increased ctDNA levels post‐treatment, designated as Group D (**Figure** **4**a). Following CRT/RT, while the concentration of cfDNA remained relatively stable (*p *= 0.395), both the mean ctDNA VAF (*p *= 0.005) and ctDNA concentration (*p *= 0.005) exhibited a significant decrease post‐treatment (Figure 4b). Patients with negative ctDNA post‐treatment exhibited significantly longer PFS (19.5 months vs 7.5 months, HR = 0.385, 95% CI: 0.208–0.713, *p *< 0.001) and OS (Not reached [NR] vs 17.4 months, HR = 0.305, 95% CI: 0.151–0.614, *p *= 0.001) (Figure 4c), highlighting the outstanding prognostic value of ctDNA detected following CRT/RT. Subgroup analysis revealed that post‐treatment ctDNA effectively predicted both PFS and OS in most subgroups (Figure S4, Supporting Information). By further stratification, the median PFS in Group A was longer than in Group B (25.6 months vs 13.9 months). However, the median OS was shorter than in Group B (29.5 months vs NR), with no significant difference overall in either PFS or OS between the two groups. Compared to Group D, Group C also did not demonstrate a significant advantage in either PFS or OS (Figure 4d). Notably, detection of ctDNA post treatment preceded radiographic progression as determined by RECIST 1.1 criteria in 75% of patients and by a median of 4.9 months. Among the test cohort patients, one patient did not undergo post‐treatment ctDNA testing, while nine out of 18 patients (50%) tested positive for ctDNA. Survival analysis revealed that patients with negative ctDNA status after treatment exhibited significantly longer OS (*p *= 0.032), with PFS also approaching statistical significance at the critical threshold (*p *= 0.051) (Figure S5a,b, Supporting Information). The borderline significance in PFS might be attributable to the limited sample size. Further subgroup analysis based on alterations in ctDNA status yielded results similar to those observed in the discovery set (Figure S5c,d, Supporting Information).

### 2.4 The Mid‐Treatment Changes in TMTV Exhibited a Strong Prognostic Value for Recurrence and Survival

A total of 33 patients underwent a second PET/CT scan during treatment and then received adaptive RT. Similar to baseline, the levels of tumor metabolic parameters during treatment could not distinguish between patients with good or poor prognosis (Table S1, Supporting Information). We further examined the association between both absolute and relative changes in these parameters from baseline and prognosis, with the data‐driven cut points determined based on the median values, except for the relative peak standardized uptake value (SUVpeak), which was categorized based on a decrease of more than 30%. The results showed that in univariate analysis, binary absolute ∆TMTV demonstrated strong predictive ability for recurrence (*p *= 0.002) and survival (*p *= 0.004) (**Table** **2**). Higher ∆SUVmax tended to be associated with longer PFS (*p *= 0.054) and OS (*p *= 0.078), but the relationship was not significant.

**Table 2:** Univariate and multivariate analyses of the prognostic significance of absolute and relative changes, including ∆TMTV, ∆TLG, ∆SUVmean, ∆SUVmax, and ∆SUVpeak.

Tumor metabolism		PFS				OS			
		Univariate analysis		Multivariate analysis		Univariate analysis		Multivariate analysis	
		HR (95% CI)	*p*	HR (95% CI)	*p*	HR (95% CI)	*p*	HR (95% CI)	*p*
Absolute^a)^	∆TMTV	0.255 (0.105–0.618)	0.002	0.278 (0.112–0.689)	0.006	0.251 (0.098–0.642)	0.004	0.269 (0.103–0.706)	0.008
	∆TLG	0.810 (0.359–1.827)	0.612			1.275 (0.539–3.014)	0.580		
	∆SUVmean	0.649 (0.284–1.482)	0.305			0.883 (0.373–2.086)	0.776		
	∆SUVmax	0.437 (0.188–1.013)	0.054	0.539 (0.220–1.318)	0.178	0.448 (0.183–1.093)	0.078	0.593 (0.234–1.502)	0.270
	∆SUVpeak	0.380 (0.164–0.884)	0.025	0.420 (0.195–1.157)	0.070	0.467 (0.191–1.139)	0.094	0.678 (0.278–1.754)	0.315
Relative^b)^	∆TMTV	0.189 (0.075–0.480)	<0.001	0.168 (0.045–0.631)	0.008	0.160 (0.059–0.435)	<0.001	0.120 (0.030–0.482)	0.003
	∆TLG	0.350 (0.150–0.816)	0.015	1.957 (0.141–27.205)	0.617	0.358 (0.146–0.876)	0.024	1.449 (0.425–4.944)	0.553
	∆SUVmean	0.363 (0.156–0.848)	0.019	0.570 (0.051–6.362)	0.648	0.512 (0.213–1.232)	0.135		
	∆SUVmax	0.564 (0.249–1.279)	0.170			0.873 (0.370–2.064)	0.758		
	∆SUVpeak	0.483 (0.210–1.109)	0.086			0.652 (0.274–1.552)	0.334		

^a)^ Absolute changes are defined as (baseline–mid‐Treatment), with the median values used as the cut‐offs;

^b)^ Relative changes are defined as (baseline–mid‐Treatment)/baseline × 100%. For relative changes of SUVpeak, the cut point 30% is based on PERCIST. For others, the data‐driven cut points at medians were selected.

Abbreviations: maximum standardized uptake value; SUVpeak, mean standardized uptake value; SUVmax, overall survival; TMTV, peak standardized uptake value, PFS, progression‐free survival; OS, total lesion glycolysis; SUVmean, total metabolic tumor volume; TLG

The results showed that both univariate and multivariate analyses of binary absolute ∆TMTV demonstrated strong predictive ability for recurrence (*p *< 0.01) and survival (*p *< 0.01) (Table 2). A higher ∆SUVpeak was significantly associated with improved PFS in the univariate analysis. Higher ∆SUVmax tended to be associated with longer PFS (*p *= 0.054) and OS (*p *= 0.078), but the relationship was not significant. The relative changes showed a more significant prognostic value. In univariate analysis, relative ∆TMTV, ∆TLG, and ∆SUVmean values above the median were all significantly associated with better survival (Table 2). However, in multivariate analysis, only ∆TMTV remained independently associated with prognosis. Unfortunately, only one patient in the test set had mid‐treatment PET scan data, so the results from the exploratory cohort could not be further validated in this section.

### 2.5 Changes in ctDNA Concentration and ∆SUVmax Were Correlated with Responses

**Figure 5:**
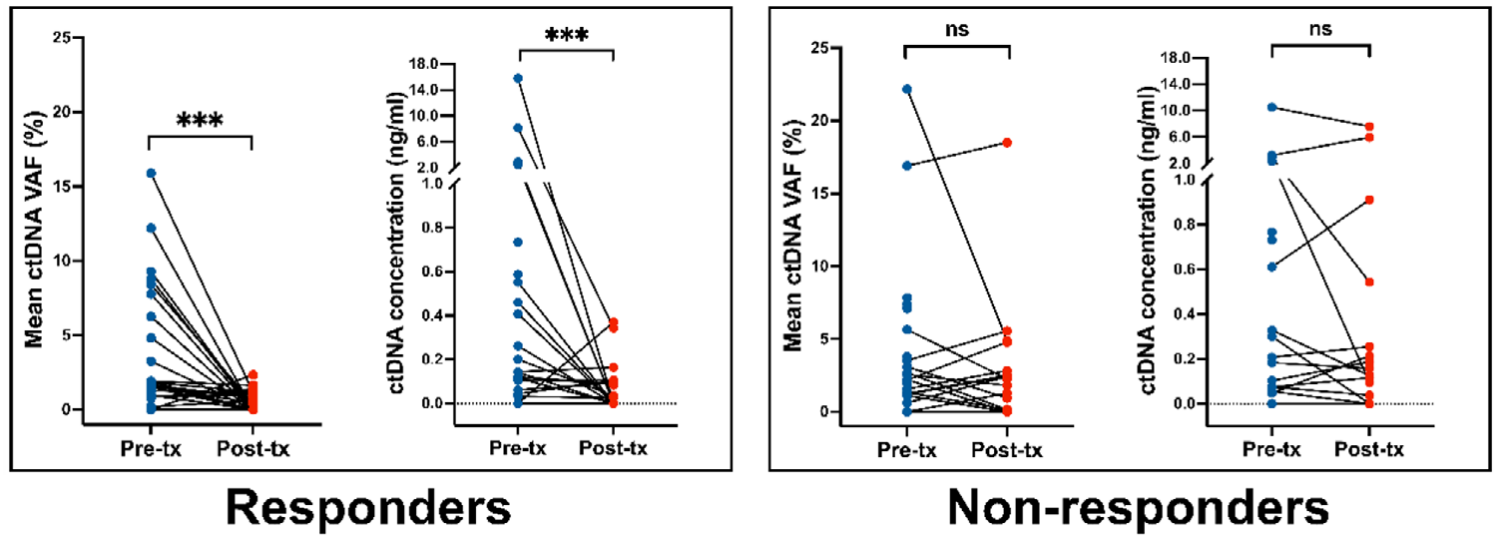
Comparison of pretreatment mean ctDNA VAF and concentration between response and non‐response groups. Abbreviations: ctDNA, circulating tumor DNA; VAF, variant allele fraction; tx, treatment; ns, not significant; *p < 0.05; **p < 0.01; ***p < 0.001.

Due to the failure of baseline information in predicting responses to treatment, we turned our hopes to the prognostic potential of changes in ctDNA VAF or concentration and PET metabolic parameters before and after treatment. Moreover, responders showed a significant decrease in both mean ctDNA VAF (*p *< 0.001) and concentration (*p *< 0.001), whereas non‐responders displayed no significant changes in the two metrics before and after treatment (**Figure** **5**). Regarding the alterations in tumor metabolic parameters, among the 33 patients undergoing mid‐treatment PET/CT, the majority exhibited varying degrees of reduction in the five parameters (Table S2, Supporting Information). Compared to the non‐response group, the response group showed significantly higher ∆SUVmax (*p *= 0.008), with a trend toward higher ∆TMTV (*p *= 0.071) and ∆TLG (*p *= 0.084), although not significant (Table S3, Supporting Information). Overall, the correlation between relative changes and absolute changes was similar.

### 2.6 Comparison of Survival in Patients with ctDNA‐/High ∆TMTV after CRT and Patients Receiving Consolidation ICI after CRT through PSM

**Figure 6:**
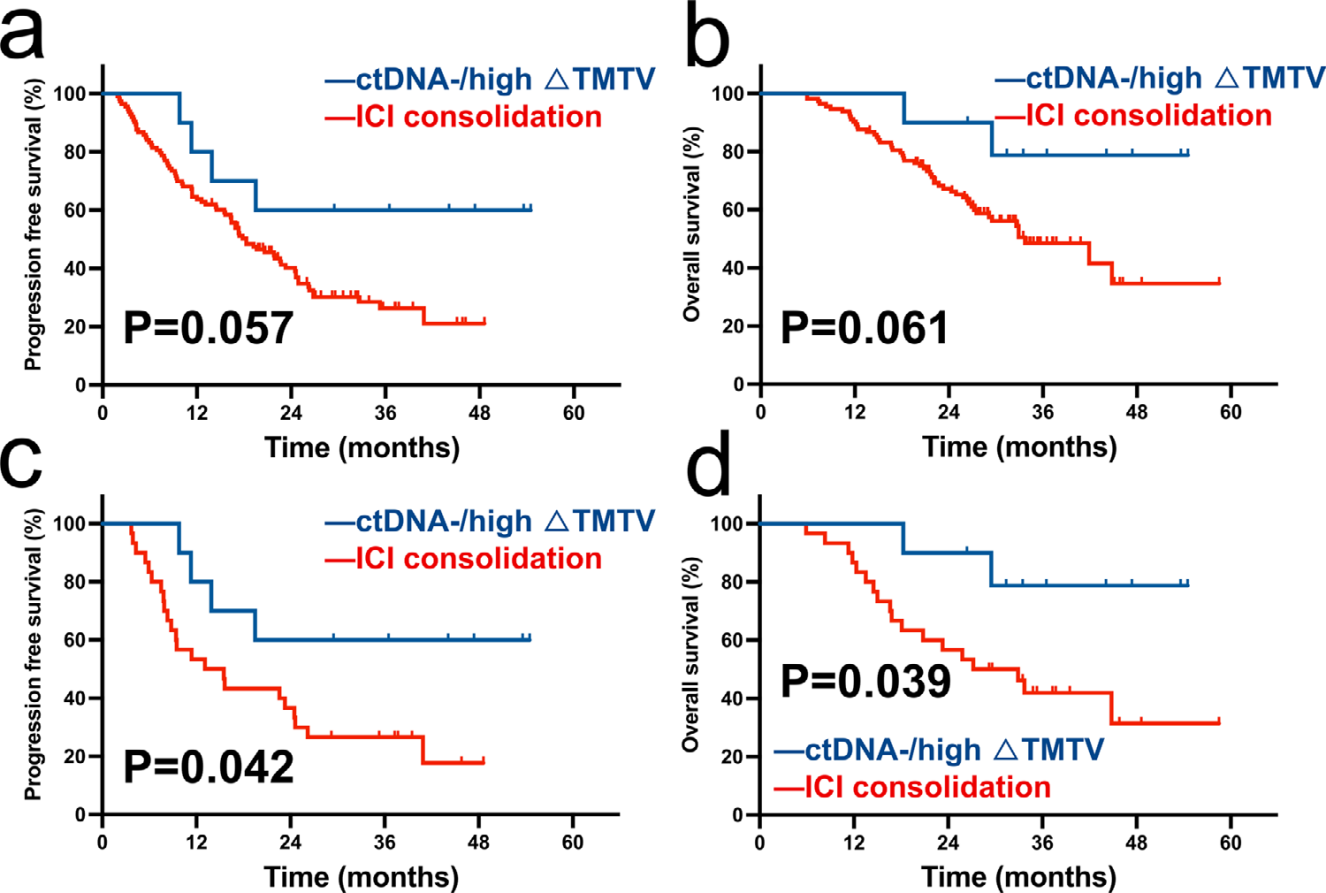
Survival comparison of PSM analysis in patients receiving ICI consolidation. a) Comparison of PFS between ctDNA‐/high ∆TMTV patients and all patients receiving ICI consolidation; b) Comparison of OS between ctDNA‐/high ∆TMTV patients and all patients receiving ICI consolidation; c) Comparison of PFS between ctDNA‐/high ∆TMTV patients and PSM patients receiving ICI consolidation; d) Comparison of OS between ctDNA‐/high ∆TMTV patients and PSM patients receiving ICI consolidation. Abbreviations: ctDNA, circulating tumor DNA; TMTV, total metabolic tumor volume; ICI, immune checkpoint inhibitor; PSM, Propensity score matching.

Consolidation ICI after definitive CRT is currently the standard treatment for unresectable LA‐NSCLC. However, some studies suggest that patients cured by radical CRT might avoid additional consolidation ICI treatment. Given the remarkable prognostic effect of post‐treatment ctDNA and ∆TMTV, we tried to determine whether patients with negative ctDNA and high ∆TMTV after CRT could forgo extra consolidation therapy. Therefore, we collected data from 113 patients with stage III unresectable LA‐NSCLC who underwent consolidation ICI after definitive CRT, with a median PFS of 18.3 months (**Figure** **6**a) and a median OS of 33.7 months (Figure 6b). We matched 10 ctDNA‐/high ∆TMTV patients after CRT with 30 patients from aforementioned ICI consolidation treatment cohort using propensity score matching (PSM) to compare survival outcomes. For unmatched patients, the survival differences between the two cohorts were not significant, although a trend was observed (Figure 6a,b). In the matched patient group, those with negative ctDNA and high ∆TMTV demonstrated significantly better PFS (NR vs 14.3 months, HR = 0.432, 95% CI: 0.193–0.969, *p *= 0.042; Figure 6c) and OS (NR vs 30.1 months, HR = 0.368, 95% CI: 0.143–0.950, *p *= 0.039; Figure 6d).

## 3 Discussion

In this study, we pioneer the investigation into the synergistic utilization of ctDNA and tumor metabolism parameters to predict clinical outcomes in unresectable LA‐NSCLC. Our findings demonstrate that ctDNA MRD analysis one‐month post‐treatment heralded disease recurrence five months ahead of imaging, establishing a robust prognostic tool. Moreover, baseline ctDNA detection in conjunction with TMTV manifested a promising prognostic role. The changes in ctDNA concentration and ∆SUVmax mirrored responses to CRT/RT. Hence, the integration of these two methodologies could augment predictive accuracy, furnishing refined guidelines for subsequent consolidation ICI.

The consensus on detectable ctDNA MRD post‐curative treatment indicating inferior survival is nearly established, yet the optimal timing, the number of testing points, and its directive significance for post‐CRT ICI continue to be debated. The initial MRD landmark analysis that garnered attention was conducted within four months post‐treatment, showing that all detectable cases progressed, while undetectable ones remained disease‐free during follow‐up.^[^ ^10^ ^]^ Subsequent research on unresectable LA‐NSCLC emphasized longitudinal dynamic ctDNA monitoring during treatment, aligning with earlier findings that post‐CRT/RT ctDNA detection holds substantial prognostic value.^[^ ^11^, ^12^ ^]^ However, potentially due to methodological variations, larger sample sizes, and extended follow‐up periods, they did not achieve such high sensitivity and specificity. Diverging levels of sensitivity and specificity were also manifested in other NSCLC ctDNA MRD studies.^[^ ^6^, ^18^ ^]^ Early ctDNA clearance, defined as negative on‐ and post‐RT, was recently proposed, underlining the significance of multi‐point dynamic monitoring.^[^ ^11^ ^]^ Such patients exhibited notably superior PFS compared to those achieving ctDNA clearance post‐RT alone. While multi‐point monitoring is more sensitive, its relatively high cost and patient compliance issues pose hurdles for its clinical application. Bi et al. analyzed data collected at five distinct time points pre‐ and post‐treatment, elucidated that ctDNA one‐month post‐CRT/RT exhibited optimal prognostic capability.^[^ ^12^ ^]^ Our investigation, focusing solely on ctDNA analysis one‐month post‐treatment, indeed revealed notable predictive power. Hence, in scenarios where dynamic monitoring is unfeasible, ctDNA detection one‐month post‐treatment could be crucial.

The potential predictive value of ctDNA MRD for consolidation ICI has also been explored.^[^ ^7^, ^11^ ^]^ Definitive CRT alone can cure some patients,^[^ ^4^, ^5^ ^]^ who could avoid unnecessary additional consolidation therapy. Patients with undetectable ctDNA after CRT exhibited favorable outcomes, regardless of consolidation ICI.^[^ ^7^ ^]^ However, similar to related literature,^[^ ^11^, ^12^ ^]^ over half of our undetected MRD cohort experienced cancer recurrence, indicating the limited sensitivity of a single time point ctDNA MRD. Non‐invasive imaging modalities may serve as supplementary predictive tools, particularly as the integration of dual‐modality approaches combining FDG‐PET and ctDNA has demonstrated complementary predictive value in other NSCLC clinical contexts.^[^ ^19^, ^20^ ^]^ The efficacy enhancement and prognostic utility of mid‐treatment PET/CT‐guided adaptive RT in LA‐NSCLC remain controversial, as evidenced by two recent large‐scale randomized trials demonstrating divergent conclusions on survival outcomes and recurrence risk stratification.^[^ ^21^, ^22^ ^]^ In our study, integrating tumor metabolic changes enabled the identification of patients with negative ctDNA and high ∆TMTV post‐treatment, whose survival outcomes paralleled those treated with the PACIFIC regimen. Therefore, integrating ctDNA monitoring with ^18^F‐FDG PET/CT could better identify those cured by CRT, averting overtreatment. Two prospective trials (NCT04585477, NCT04585490) are evaluating treatment adjustments based on ctDNA after CRT, aiming to guide treatment intensification or de‐escalation. Meanwhile, the LUARA study has ushered in a new era of EGFR‐TKI consolidation for *EGFR*‐mutant, unresectable NSCLC,^[^ ^23^ ^]^ and the role of ctDNA and PET parameters in guiding consolidation therapy for these patients requires further investigation.

Currently, the prognostic significance of pre‐treatment ctDNA levels, their influence on clinical decision‐making is unclear. Several studies on NSCLC RT/CRT failed to differentiate outcomes based on baseline ctDNA, a recent study in localized esophageal squamous cell carcinoma also found no significant correlation between baseline ctDNA, PFS, and OS.^[^ ^24^ ^]^ However, multiple studies across various cancers have shown a favorable prognostic role for preoperative ctDNA,^[^ ^18^, ^25^, ^26^, ^27^ ^]^ especially highlighted by recent PROPHET ctDNA analysis.^[^ ^27^ ^]^ We hypothesize that the reduced predictive value of ctDNA before CRT/RT could stem from variations in detection methods, advanced staging, or potential resistance to CRT. Notably, utilizing ^18^F‐FDG PET/CT scans at diagnosis in our study has improved the predictive accuracy of baseline data. Similarly, a recent study established an imaging framework utilizing pre‐treatment CT, PET/CT, demonstrating complementary value to pre‐, post‐treatment ctDNA analysis in predicting disease recurrence after curative surgery or radiotherapy.^[^ ^20^ ^]^ Moreover, RT‐induced pulmonary fibrosis poses a challenge in differentiating residual or recurrent disease on cross‐sectional imaging.^[^ ^10^, ^28^ ^]^ Although the measurement of ctDNA MRD emerged as a discerning factor for equivocal imaging, demonstrating high sensitivity, specificity in predicting recurrence, post‐treatment ctDNA status does not reflect treatment efficacy. Upon further analysis, in terms of quantitative diagnosis, we discovered a correlation between the changes in ctDNA concentration, ΔSUVmax in relation to treatment response. Similarly, Li et al. identified that post‐treatment alterations in ctDNA levels could serve as a robust predictor for early treatment response.^[^ ^29^ ^]^ Thus, beyond the ability of post‐treatment ctDNA status to precede radiographic progression, the integration of ctDNA changes, ΔSUVmax holds the potential to augment the analysis of short‐term response assessment, surveillance imaging, aiding in the interpretation of equivocal scans.

The limitations of our study should be acknowledged. First, the limited sample size and the inclusion of only two landmark time points restricted our ability to perform dynamic ctDNA monitoring, thereby precluding analysis of ctDNA dynamics during radiographic progression. Second, the unavailability of baseline primary tumor tissue samples prevented comparative genomic profiling between tissue biopsies and liquid biopsies, limiting insights into tumor heterogeneity evolution. Third, heterogeneity arising from diverse baseline characteristics and combined treatment modalities (CRT/RT) may have introduced confounding effects, potentially influencing the analytical results. Fourth, due to the early design of this study, some potentially meaningful parameters, such as the SUVpeak corrected for lean body mass (SULpeak)^[^ ^30^, ^31^, ^32^, ^33^ ^]^ and blood tumor mutational burden,^[^ ^34^ ^]^ were not included, limiting the comprehensiveness of the analysis. While SUVpeak refers to the maximum standardized uptake value (SUV) value within the region of interest, SULpeak normalizes SUVpeak by lean body mass. Incorporating SULpeak into prognostic analysis may further enhance its reliability, providing greater consistency across patients and improving the accuracy of therapy response assessment, especially in individuals with varying body compositions.^[^ ^30^, ^31^, ^32^, ^33^ ^]^

Finally, while the integration of dual‐modality monitoring enhanced predictive capability, our study does not provide novel methodological insights or significant technical advancements. Future studies incorporating multiomics approaches into personalized detection frameworks are warranted to refine predictive precision and address these unresolved questions.

## 4 Conclusion

This study demonstrated the strong prognostic role of post‐treatment ctDNA and the limitations of baseline ctDNA as an effective biomarker while highlighting the complementary predictive value of metabolic parameters. Combining baseline ctDNA with TMTV enhances survival predictions, and ∆TMTV alone strongly predicts recurrence and survival. Regarding the prediction of CRT/RT efficacy, changes in ctDNA concentration and ΔSUVmax were correlated with treatment responses. Notably, patients with undetectable ctDNA and high ∆TMTV post‐CRT/RT even demonstrated improved PFS and OS compared to those who received ICI consolidation therapy, suggesting that CRT alone could be sufficient for these patients, thus avoiding unnecessary ICI therapy. These findings underscore the predictive efficacy of integrating ctDNA with metabolic parameters, particularly in providing potential guidance for consolidation therapy.

## 5 Experimental Section

### Study Design and Patients’ Recruitment

The blood samples for this prospective study based on ctDNA detection were obtained from patients enrolled in a phase II randomized trial (ChiCTR2400081896),^[^ ^35^ ^]^ which was supplementarily registered in 2024, as well as from subsequent samples collected for the observational study. Patients diagnosed with stage III unresectable NSCLC treated with definitive CRT/RT from November 2012 to April 2019 at the Zhejiang Cancer Hospital were recruited, either with or without ^18^F‐FDG PET/CT adapted RT. This study was approved by the Ethics Review Committee of Zhejiang Cancer hospital (No. IRB2012‐115). In addition, 9 patients from Shanghai Pulmonary Hospital (approved by Ethics Committee of Shanghai Pulmonary Hospital, No. 19228FL), who were enrolled in the same manner as in this study, and 10 patients from Shandong Cancer Hospital (approved by Ethics Committee of Shandong Cancer Hospital, No. SDTHEC201803033), who underwent serial ctDNA monitoring using the same panel during definitive CRT for stage III NSCLC, were included in the test set. All these patients also received ^18^F‐FDG PET/CT scans prior to treatment. All participating patients provided written informed consent. The conduct of this study adhered to the principles outlined in the Declaration of Helsinki.

### Treatment Procedure

In this observational, non‐interventional study, all patients underwent a standard treatment protocol, involving concurrent or sequential CRT. RT alone was administered to those who could not tolerate chemotherapy. The chemotherapy regimen consisted of a platinum‐based doublet chemotherapy. All patients underwent an ^18^F‐FDG PET/CT scan prior to the commencement of treatment. Among them, 33 patients received PET/CT guided adaptive RT due to their enrollment in the randomized controlled trial.^[^ ^35^ ^]^ RT was conventionally delivered in 2 Gy per fraction, with the planning target volume (PTV) receiving a total dose of 54–70 Gy. After 20 fractions, with the PTV dose reaching 40 Gy, a secondary CT simulation was executed in the initial setup position for all patients. For those receiving PET/CT guided adaptive RT, an additional mid‐treatment ^18^F‐FDG PET/CT scan was performed. The subsequent RT plan was adjusted based on the imaging changes observed during treatment, as shown by the mid‐treatment PET/CT and CT scans. The gross tumor volume (GTV) was automatically outlined as 1.5 times the ^18^F‐FDG PET/CT SUV of the aortic blood pool. PET/CT data were imported into the RayStation treatment planning system (RaySearch Laboratories, Stockholm, Sweden) in DICOM format, with the GTV subsequently adjusted based on the contrast‐enhanced CT (CE‐CT) simulation scan. In the conventional treatment arm, the target area was manually contoured in RayStation based on the CE‐CT simulation scan. All patients were administered an intensity‐modulated radiation therapy regimen, endeavoring for 95% of the PTV to attain the entire prescribed dose. Dosimetry to other normal structures was meticulously orchestrated within the ambit of standard practice, in stringent adherence to the National Comprehensive Cancer Network guidelines.

### PET/CT Protocol and Data Analysis

The ^18^F‐FDG PET/CT scanning was executed at the Department of Nuclear Medicine of Zhejiang Cancer Hospital. Two experienced nuclear medicine specialists performed ^18^F‐FDG PET/CT scans following a standardized protocol. Following a 6‐hour fasting period, FDG (3.7 MBq [0.1 mCi] kg^−1^) was intravenously administered, ensuring a blood glucose level of below 11 mmol L^−1^. A 60‐minute interval was observed between the injection and the commencement of scanning. Each patient underwent a 5‐minute whole‐body emission scan covering the area from the base of the skull to the mid‐femur. During the scan, patients were instructed to maintain a calm demeanor and adhere to slow breathing. The ^18^F‐FDG PET/CT scanning was performed utilizing a Discovery 710 PET/CT scanner (GE Medical Systems, Milwaukee, Wisconsin, USA), with an axial slice thickness of 4.25 mm. The acquired multi‐slice CT images were employed for attenuation correction and subsequent reconstruction. Display of the PET, CT, and integrated PET/CT images in coronal, sagittal, and transverse planes was facilitated on a Xeleris workstation (GE Healthcare)

PET/CT images from both baseline and mid‐treatment were analyzed using MIM software (version 7.1.7, Cleveland, OH, USA). A threshold of SUV 2.5 was applied to automatically identify and define regions of interest around hypermetabolic lesions. All lesions with significant FDG uptake were included in the analysis, while lesions exhibiting physiological or inflammatory hypermetabolism were excluded. The SUVmax, SUVpeak, SUVmean, TLG, and metabolic tumor volume (MTV) for primary tumors and mediastinal lymph nodes were measured using MIM software. The TMTV was derived by summing the MTV values of all lesions. The absolute changes in these parameters were defined as the baseline value minus the value during treatment, for example, ∆TMTV = baseline TMTV–mid‐treatment TMTV. The relative changes were the ratio of the absolute changes to the baseline value. For the binary marker, in addition to categorizing relative ∆SUVpeak based on a 30% decrease,^[^ ^30^ ^]^ the cutoff points were determined based on the median values derived from the data

### Sample Collection and Preservation

In this study, baseline blood samples were collected up to 3 days before CRT/RT. The landmark time point of post‐treatment was defined as 1 month (±3 days) after CRT/RT. Collected 8–10 mL of venous blood into an EDTA‐K2 anticoagulant blood collection tube, and gently invert the tube several times to ensure thorough mixing. Within 2 h, separate out plasma samples by centrifuging at 1800 g for 10 min. The plasma sample was transferred into a 5 mL centrifuge tube. After centrifugation, ensured a remaining whole blood sample of no less than 0.5 mL, transferred to another centrifuge tube. Labeled the plasma/whole blood samples, transported at room temperature, and stored at −80 °C until testing.

### DNA Extraction and Library Preparation

The separated plasma fraction of peripheral blood samples were subjected to cfDNA extraction using the Qiagen QIAamp Circulating Nucleic Acid Kit (Qiagen, Dusseldorf, Germany). Purified cfDNA was assessed for quality using a Nanodrop2000 spectrophotometer (Thermo Fisher Scientific, Waltham, MA) and quantified with a Qubit 2.0 fluorometer (Life Technologies, Waltham, MA) using the dsDNA HS Assay Kit. Approximately 50 ng of cfDNA underwent end repair, A‐tailing, and ligation with indexed adapters following the optimized protocol of the KAPA Hyper Prep Kit for library preparation. The samples then underwent size selection using Agencourt AMPure XP beads (Beckman Coulter, Mississauga, Canada), followed by polymerase chain reaction amplification with the KAPA Hyper DNA Library Prep Kit (KAPA Biosystems, Wilmington, MA). To investigate tumor‐driving genes, a DNA probe library designed to hybridize with the target genes was employed. Target enrichment was performed using custom‐designed xGen Lockdown probes (Integrated DNA Technologies), targeting 474 cancer‐ and RT‐related genes (Radiotron, Nanjing Geneseeq Technology Inc., Nanjing). This panel, with a sequencing length of 150 base pairs, was used in several published studies.^[^ ^12^, ^34^ ^]^

### Machine Sequencing and Bioinformatics Analysis

The post‐enrichment libraries were sequenced using the Illumina HiSeq sequencing platform with a PE150 kit. An average sequencing depth of not less than 1000× was ensured for tissue samples, not less than 5000× for cfDNA samples, and not less than 100× for whole blood control samples.

The sequencing output data was in bcl format, converted it to FASTQ (version: illumina 1.8+) format using bcl2fastq (v2.17.1.14, Illumina, Inc.) software. Data quality control was performed using Trimmomatic, and the post‐quality control data was aligned to the human reference genome (hg19, GRCh37 Genome Reference Consortium Human Reference 37) using BWA (0.7.12) MEM algorithm with default parameters, generating SAM files. Picard (1.119) was used to convert SAM files to BAM files and sorted them based on chromosome coordinates. Used Genome Analysis Toolkit (GATK, version 3.4‐0) to optimize the alignment results of local alignment. For the analysis of single nucleotide variants and Insertions/Deletions (Indels), VarScan2 software (2.3.9) was used, the minimum detection frequency was set to 0.01 and the *p*‐value to 0.05, generating VCF results. The VCF results were annotated with ANNOVAR and further manually inspected the results with IGV. ADTEx software (1.0.4) was employed for copy number variations analysis.

### Follow Up

Patients were initially followed up at a one‐month interval after treatment, then quarterly for three years, semi‐annually for the subsequent two years, and annually thereafter. The terminal follow‐up was conducted on March 10, 2023. The follow‐up evaluations encompassed contrast‐enhanced CT scans of the thoracic and abdominal regions, ultrasonographic examination of the cervical and supraclavicular lymph nodes, alongside routine hematological assessments. PFS was delineated from the first day of treatment to the incidence of tumor progression (inclusive of local recurrence, nodal recurrence, or distant metastases), death, or the last follow‐up. OS was demarcated from the onset of treatment to death or censored at the last follow‐up. Annual evaluations for distant metastases consisted of bone scans and brain magnetic resonance imaging. Treatment responses were evaluated from the baseline, using the response evaluation criteria in solid tumors (RECIST), version 1.1, as the assessment modality.

### Statistical Analysis

For baseline demographic and disease characteristics, continuous variables were summarized as medians, ranges, means, and SD, and categorical data were analyzed as proportions. Correlation analysis and regression analysis were used to examine the relationship between ctDNA concentration and metabolic parameters. In correlation analysis, Pearson correlation was used for data that follow a normal distribution, while Spearman correlation was used for data that did not follow a normal distribution. For normally distributed data, either a paired *t*‐test or unpaired *t‐*test could be used to analyze differences between two samples. For non‐normally distributed data, the Mann–Whitney *U*‐test was used to analyze differences between two independent samples, while the Wilcoxon signed‐rank test was used for paired samples. PSM was used to match patients from an ICI consolidation cohort with patients from a subgroup from this study whose baseline characteristics were similar. PFS and OS were estimated using the Kaplan–Meier method. Medians with 95% confidence intervals (CIs) were calculated along with survival probabilities. Cox proportional hazards regression models were conducted to correlate ctDNA status and other variables of interest with survival. Hazard ratios (HR) and 95% CIs were generated. Statistical significance was defined as a *p‐*value <0.05 (two‐sided), with a significance level (*α*) of 0.05 for all tests. All the data were analyzed using SPSS version 23.0 (IBM Corp., Armonk, NY, USA), GraphPad Prism 10.4.0, and R programming.

## Conflict of Interest

The authors declare no conflict of interest.

## Author Contributions

L.W., Z.Z., C.J., X.S., and L.L. contributed equally to this work. L.W., Z.Z., and M.B. analyzed the data, drafted the manuscript, and prepared the figures and tables. Y.Y. and M.L. revised the manuscript. C.J., X.S., and L.L. were responsible for patient enrollment, blood sample collection, and processing, as well as the collection and organization of patient PET scan images and data. K.X. conducted quality control and testing of the blood samples. Y.X., J.Y., S.Y., and J.S. designed the experiments and provided resources to conduct the study. All authors have read and approved the final manuscript.

## Supporting information



Supporting Information

## Data Availability

The data that support the findings of this study are available from the corresponding author upon reasonable request.
