# Correction to “[Integration of Circulating Tumor DNA and Metabolic Parameters on ^18^F‐Fludeoxyglucose Positron Emission Tomography for Outcome Prediction in Unresectable Locally Advanced Non‐Small Cell Lung Cancer]”

**DOI:** 10.1002/advs.202506627

**Published:** 2025-05-20

[Leilei Wu; Zhenshan Zhang; Chenxue Jiang; Li Li; Xiaojiang Sun; Menglin Bai; Ming Liu; Kangli Xiong; Jinbiao Shang; Jinming Yu; Shuanghu Yuan; Yang Yang; Yaping Xu; Integration of circulating tumor DNA and metabolic parameters on ^18^F‐Fludeoxyglucose positron emission tomography for outcome prediction in unresectable locally advanced non‐small cell lung cancer, ***Adv Sci***, 2025, 12(13): e202413125.]

[In paragraph 1 of the “Experimental Section” section, the text “This study was approved by the Ethics Review Committee of Zhejiang Cancer Hospital (No. IRB2012‐115).” was incorrect. This should have read: “This study was approved by the Ethics Review Committee of Zhejiang Cancer Hospital (No. IRB‐2016‐115, with a supplementary approval granted in 2016). Subsequent samples collected after July 2017 were also approved by the Ethics Review Committee of Zhejiang Cancer Hospital (No. IRB‐2017‐195).”]

We apologize for this error.

