# Supplementary material for: Integration of Circulating Tumor DNA and Metabolic Parameters on 18F‐Fludeoxyglucose Positron Emission Tomography for Outcome Prediction in Unresectable Locally Advanced Non‐Small Cell Lung Cancer
Source: Adv Sci (Weinh). 2025 Mar 16;12(13):2413125. doi: 10.1002/advs.202413125 (PMC11967874; doi:10.1002/advs.202413125)
Supplement: Supplementary file 1 — Supporting Information [file ADVS-12-2413125-s001.docx]

Supporting Information

Integration of circulating tumor DNA and metabolic parameters on ^18^F-fludeoxyglucose positron emission tomography for outcome prediction in unresectable locally advanced non-small cell lung cancer

*Leilei Wu, Zhenshan Zhang, Chenxue Jiang, Li Li, Xiaojiang Sun, Menglin Bai, Ming Liu, Kangli Xiong, Jinbiao Shang, Jinming Yu^*^, Shuanghu Yuan^*^, Yang Yang^*^, Yaping Xu^*^*

((Please insert your Supporting Information text/figures here. Please note: Supporting Display items, should be referred to as Figure S1, Equation S2, etc., in the main text…)

**
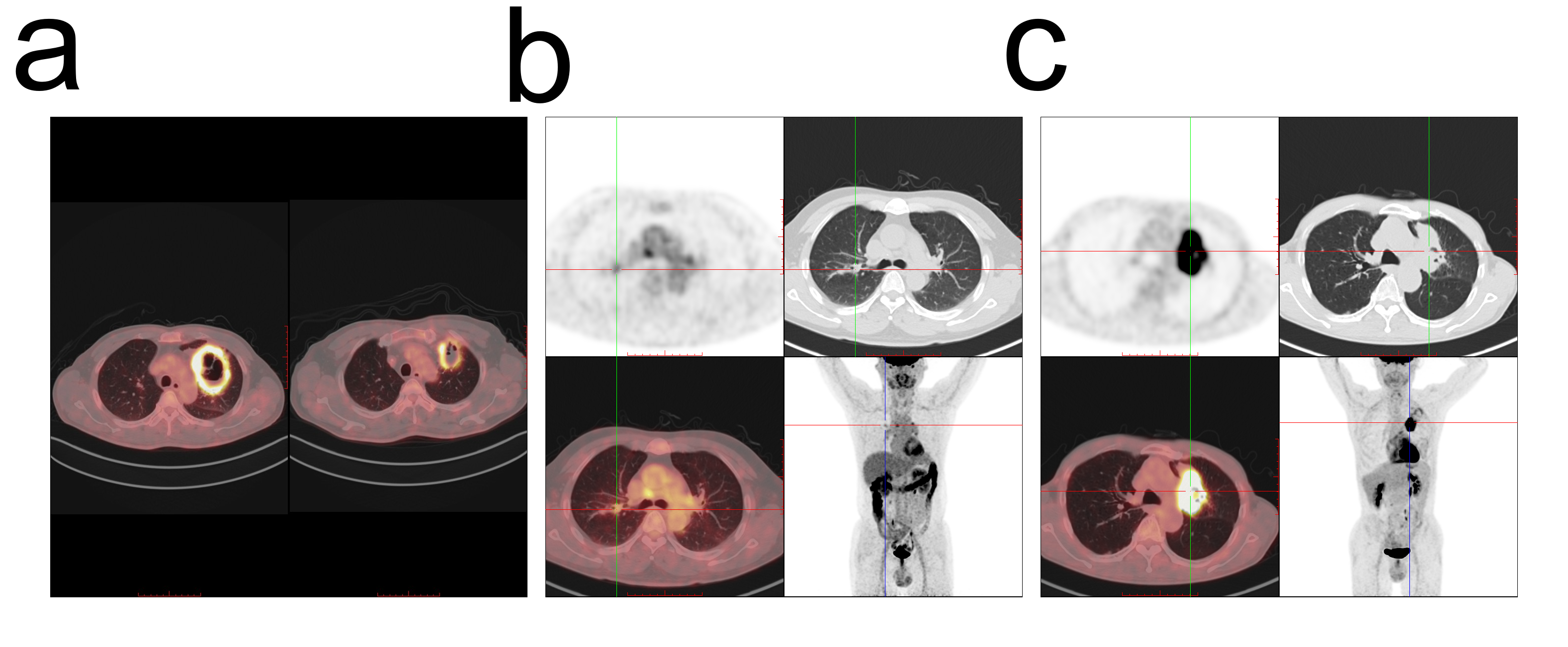
**

**Figure S1.** a) PET images of a locally advanced adenocarcinoma (cT4N2M0) before and after chemoradiotherapy, with a baseline SUVmax of 16.45 and a post-treatment SUVmax of 8.22. b,c) The detailed images of two patients in Figure 2a, with the higher SUVmax being 17.02 and the lower SUVmax being 2.54.


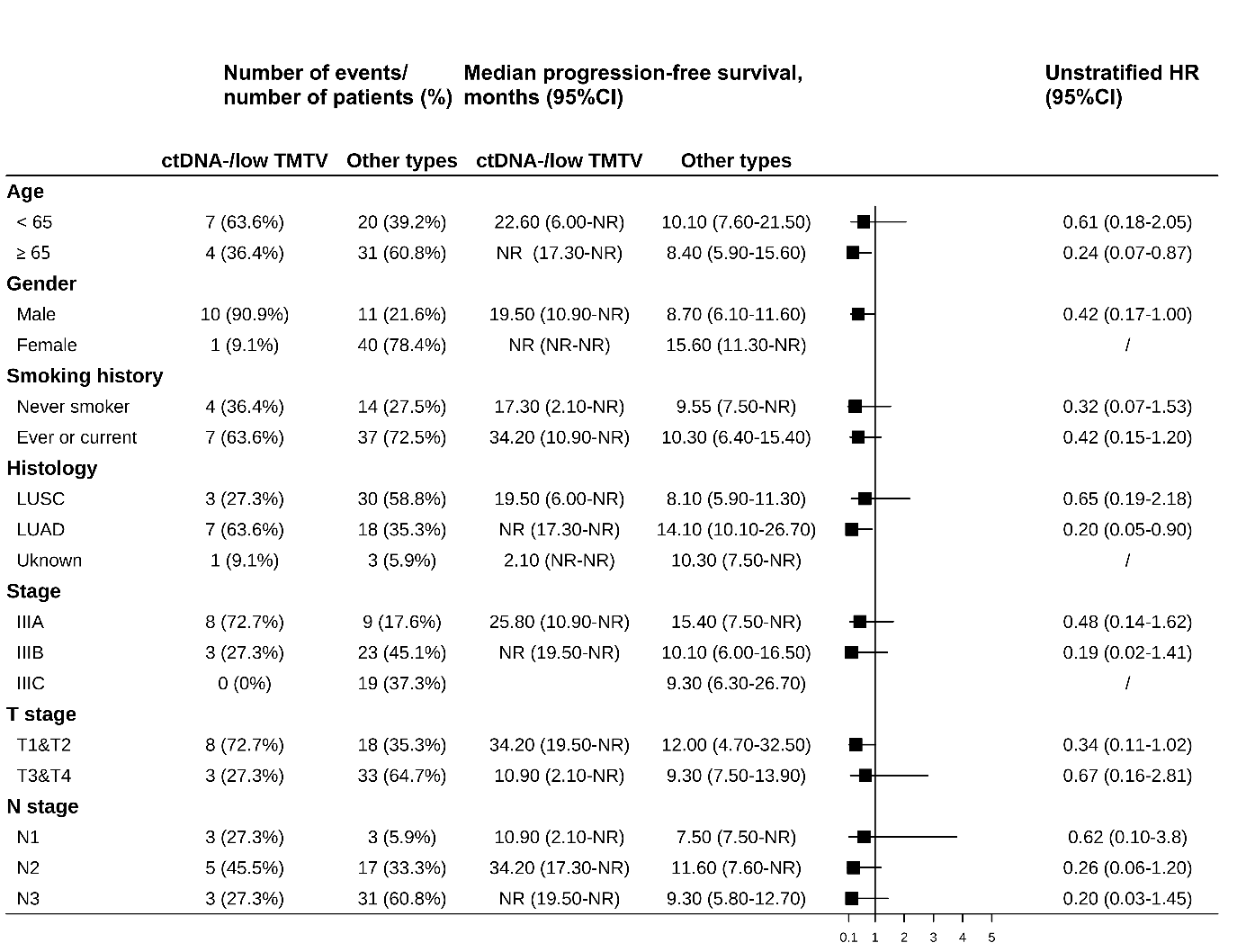


**Figure S2.** Subgroup analysis of baseline ctDNA-/low TMTV as a predictor of progression-free survival. Abbreviations: HR, hazard ratio; NR, not reached; ctDNA, circulating tumor DNA; LUSC, lung squamous cell carcinoma; LUAD, lung adenocarcinoma.

**
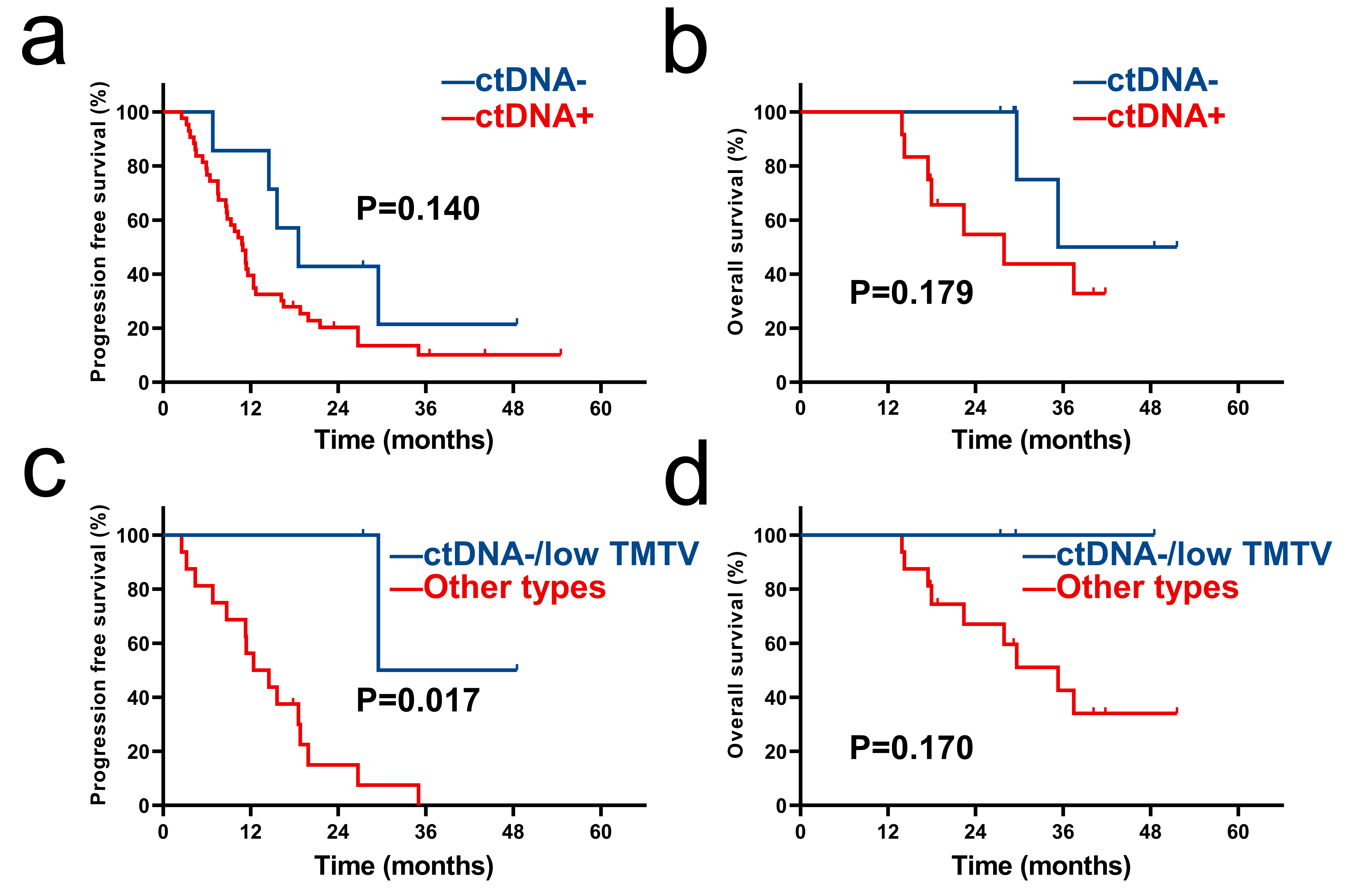
**

**Figure S3.** a,b) Kaplan-Meier curves for PFS and OS stratified by baseline ctDNA status in the test set. c,d) Kaplan-Meier curves for PFS and OS, comparing ctDNA-/TMTV low group with other types in the test set. Abbreviations: CRT, chemoradiotherapy; ctDNA, circulating tumor DNA; TMTV, total metabolic tumor volume.


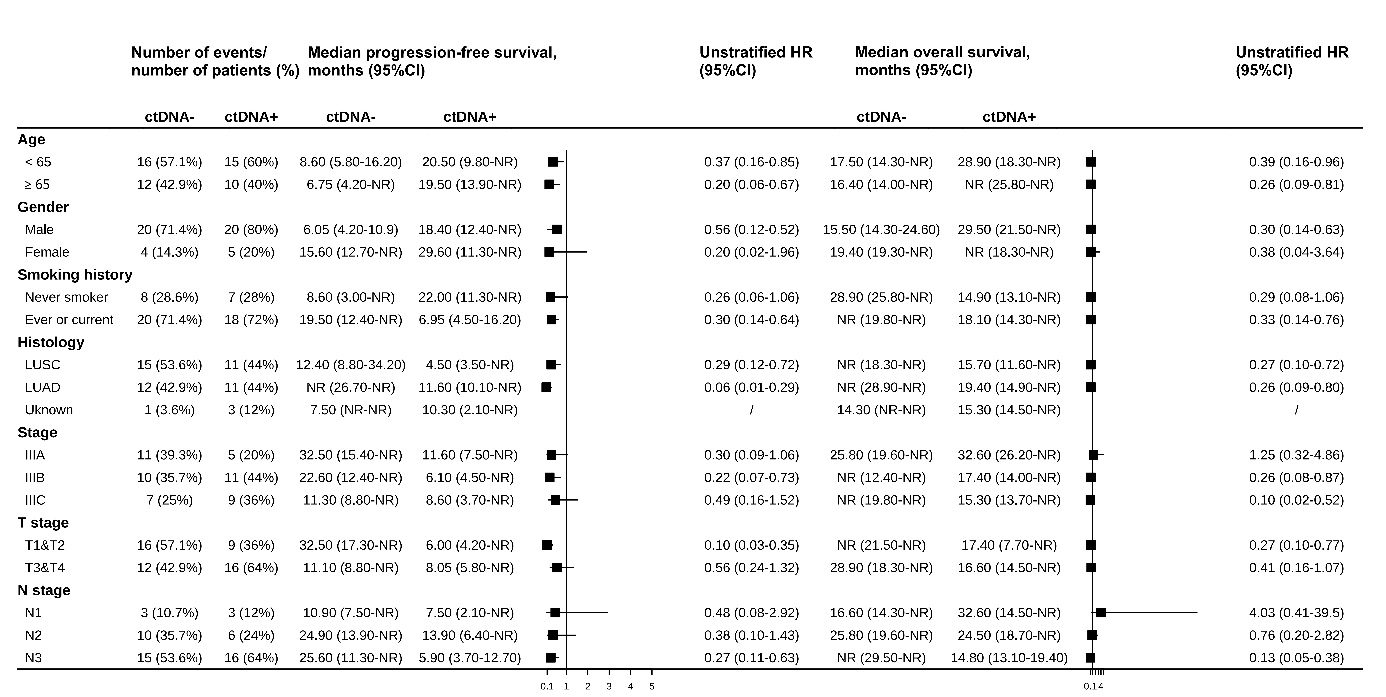


**Figure S4.** Subgroup analysis of post-treatment negative ctDNA as a predictor of progression-free survival and overall survival. Abbreviations: HR, hazard ratio; NR, not reached; ctDNA, circulating tumor DNA; LUSC, lung squamous cell carcinoma; LUAD, lung adenocarcinoma.

**
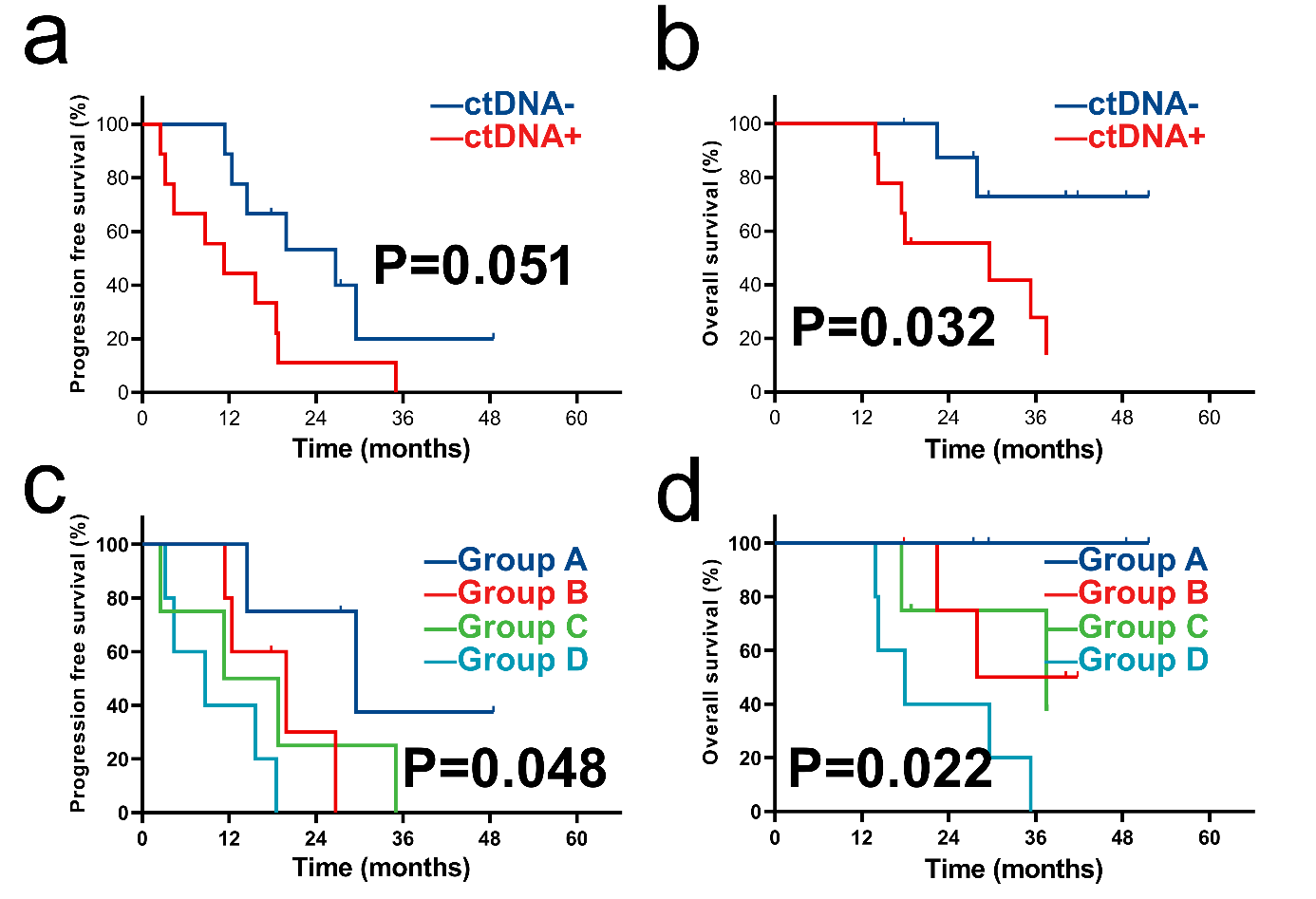
**

**Figure S5.** a,b) Kaplan-Meier curves for PFS and OS stratified by post-treatment ctDNA status in the test set. c,d) Kaplan-Meier curves for PFS and OS further stratified by pre- and post-treatment ctDNA status in the test set. Abbreviations: ctDNA, circulating tumor DNA; PFS, progression-free survival; OS, overall survival.

**Table S1 Univariate analyses of the prognostic significance of baseline and mid-treatment TMTV, TLG, SUVmean, SUVmax, and SUVpeak for PFS and OS.**

| **Tumor metabolism** | | **PFS** | | **OS** | |
| --- | --- | --- | --- | --- | --- |
|  |  | **HR (95% CI)** | ***p*** | **HR (95% CI)** | ***p*** |
| Baseline | TMTV | 0.689 (0.391-1.213) | 0.197 | 0.886 (0.483-1.624) | 0.695 |
|  | TLG | 0.792 (0.451-1.392) | 0.418 | 0.877 (0.478-1.608) | 0.671 |
|  | SUVmean | 0.866 (0.494-1.518) | 0.616 | 0.911 (0.497-1.671) | 0.763 |
|  | SUVmax | 0.815 (0.464-1.431) | 0.477 | 0.714 (0.389-1.309) | 0.276 |
|  | SUVpeak | 0.769 (0.438-1.350) | 0.360 | 0.831 (0.452-1.526) | 0.550 |
| Mid-treatment | TMTV | 0.508 (0.223-1.155) | 0.106 | 0.681 (0.440-1.054) | 0.085 |
|  | TLG | 0.541 (0.239-1.224) | 0.140 | 0.754 (0.490-1.160) | 0.198 |
|  | SUVmean | 1.056 (0.471-2.364) | 0.895 | 1.374 (0.568-3.324) | 0.481 |
|  | SUVmax | 0.730 (0.327-1.630) | 0.442 | 0.890 (0.373-2.123) | 0.793 |
|  | SUVpeak | 0.678 (0.302-1.519) | 0.345 | 0.824 (0.535-1.267) | 0.378 |

Abbreviations: PFS, progression-free survival; OS, overall survival; TMTV, total metabolic tumor volume; TLG, total lesion glycolysis; SUVmean, mean standardized uptake value; SUVmax, maximum standardized uptake value, SUVpeak, peak standardized uptake value.

**Table S2 Comparison of metabolic parameters during and before treatment.**

| Tumor metabolism | Decrease (n) | Increase (n) | *p* |
| --- | --- | --- | --- |
| TMTV | 24 | 9 | 0.036 |
| TLG | 29 | 4 | <0.001 |
| SUVmean | 30 | 3 | <0.001 |
| SUVmax | 31 | 2 | <0.001 |
| SUVpeak | 30 | 3 | <0.001 |

Abbreviations: TMTV, total metabolic tumor volume; TLG, total lesion glycolysis; SUVmean, mean standardized uptake value; SUVmax, maximum standardized uptake value, SUVpeak, peak standardized uptake value.

**Table S3 Comparison of ∆TMTV, ∆TLG, ∆SUVmean, ∆SUVmax and SUVpeak between response and non-response groups.**

|  | Tumor metabolism | *p* |
| --- | --- | --- |
| Absolute | ∆TMTV | 0.071 |
|  | ∆TLG | 0.084 |
|  | ∆SUVmean | 0.142 |
|  | ∆SUVmax | 0.008 |
|  | ∆SUVpeak | 0.029 |
| Relative | ∆TMTV | 0.128 |
|  | ∆TLG | 0.079 |
|  | ∆SUVmean | 0.196 |
|  | ∆SUVmax | 0.005 |
|  | ∆SUVpeak | 0.016 |

Abbreviations: TMTV, total metabolic tumor volume; TLG, total lesion glycolysis; SUVmean, mean standardized uptake value; SUVmax, maximum standardized uptake value, SUVpeak, peak standardized uptake value.
